# Group B Streptococcus Meningitis in a Child with Cochlear Implant

**DOI:** 10.3201/eid1510.081243

**Published:** 2009-10

**Authors:** Daniel Glikman, Michal Luntz, Rabia Shihada, Zeev Zonis, Lea Even

**Affiliations:** Western Galilee Hospital, Nahariya, Israel (D. Glikman, Z. Zonis, L. Even; Bnai-Zion Medical Center, Haifa, Israel (M. Luntz, R. Shihada); Technion-Israel Institute of Technology, Haifa (M. Luntz, L. Even)

**Keywords:** Group B Streptococcus, meningitis, cochlear implant, bacteria, streptococci, letter

**To the Editor:** *Streptococcus* *agalactiae*, designated group B streptococcus (GBS), is a major cause of infections in neonates and young infants (*1*). Invasive GBS disease in children beyond infancy is uncommon, occurring mainly as bacteremia without a focus; meningitis caused by GBS is rarely reported (*2*). Cochlear implant recipients have been documented as having a higher rate of postimplantation bacterial meningitis than a cohort of the same age in the general US population (*3*). However, no cochlear implant recipient described has been reported to be infected with GBS. We report a case of GBS meningitis in a 6-year-old boy with a cochlear implant.

The patient was hospitalized in 2007 with a 1-day history of fever, headache, and vomiting. His medical history indicated congenital bilateral deafness diagnosed at 1 month of age and consistent with Patterson syndrome (i.e., unusual facies, deafness, bronzed hyperpigmentation of the skin, cutis laxa, mental retardation, and bony deformities) (*4*). At 4 years of age, he received a right-ear cochlear implant with good functional result. Preoperative high-resolution computed tomography of the temporal bones showed bilateral inner ear malformations of both the cochlear and vestibular labyrinth, conditions consistent with bilateral Mondini deformity (*5*). Mastoids and middle ears were well aerated. No evidence of cerebrospinal fluid leak appeared during physical examination or imaging. He received a dose of 23-valent pneumococcal polysaccharide vaccine.

At the time of hospital admission, he was somnolent but could be aroused and was cooperative. He had nuchal rigidity, dysmorphic facies, and oligodactyly. Fundi, skin, and ears were unremarkable on examination. Lumbar puncture showed a total protein level of 204 mg/dL, a glucose level of 1.6 mmol/L (blood glucose 3 mmol/L), and 4,800 leukocytes/mm^3^ with 88% neutrophils; no bacteria were seen on the Gram stain. Blood count was remarkable for leukocytosis of 30,000/mm^3^ and neutrophil predominance.

The patient received treatment with dexamethasone, vancomycin, and ceftriaxone; after treatment, his condition improved rapidly. Blood culture was sterile, but GBS grew in the cerebrospinal fluid culture (the isolate being resistant only to tetracycline). Therapy was continued with ampicillin for 3 weeks. Repeated testing of his hearing and speech perception with the cochlear implant showed no deterioration.

GBS plays a major role in early- and late-onset infections in neonates and young infants (*1*). Infections in older children and adults have been described, especially in elderly patients or those suffering from chronic conditions such as diabetes mellitus, malignancy, or HIV infection (*6*). A review of medical records of patients with GBS infections over a 7-year period at a children’s hospital in Memphis, Tennessee, USA, showed that, among 18 patients >3 months of age (13% of all GBS infections in the study), bacteremia was most commonly reported; 3 cases of ventriculo-peritoneal shunt infections were recorded, but no cases of meningitis without foreign devices were found (*2*). GBS meningitis in children beyond infancy is rare; only a few cases have been reported (*7*).

Cochlear implantation is the standard treatment for children and adults affected by severe and severe-to-profound sensorineural hearing loss. The implant is a neural stimulator with an electrode array surgically placed near the auditory nerve fibers in the scala tympani of the cochlea. Pediatric cochlear implant recipients were found to be at higher risk for developing bacterial meningitis than children in the general US population (*3*). Increased risk was evident in the perioperative period but extended to >2 years postimplantation (*8*). Most meningitis cases were associated with an implant with a positioner, a silastic wedge inserted next to the implanted electrode in the cochlea to position the electrode closer to the cochlear nerve endings and thus facilitate electrical signal transmission. Most of those infections were caused by *Streptococcus pneumoniae*, and none by GBS (*3*,*8*). In our patient, the implant did not include a positioner. The timing of meningitis was consistent with the timing indicated in previous reports, but the infecting organism was unique.

Inner ear malformations themselves are associated with increased risk for meningitis (*9*). The patient reported here had bilateral inner ear malformations; therefore, estimating the relative role of the deformity compared with the cochlear implant’s role in the pathogenesis of meningitis in his case is difficult. Meningitis in patients with inner ear malformations is associated with bacteria (e.g., *S. pneumoniae* and *Haemophilus influenzae*) that colonize the upper airways. The prevalence of oropharyngeal colonization with GBS is low (≈5%), explaining the rarity of GBS meningitis (*10*). Unlike for pneumococcal meningitis, which can be prevented at least partially by vaccination, no vaccine is available for GBS.

Our report adds another example to the growing spectrum of invasive GBS disease beyond infancy. GBS is uniformly susceptible to penicillin; therefore, treatment directed at common causes of bacterial meningitis is also appropriate for GBS (*1*,*10*). Cochlear implant recipients with symptoms of fever, otitis media, or headache should be carefully assessed; if meningitis is diagnosed, GBS should be considered as a possible causative organism.
